# Disability during the last ten years of life: evidence from a register-based study in Austria

**DOI:** 10.1007/s10433-024-00823-z

**Published:** 2024-09-28

**Authors:** Erwin Stolz, Anna Schultz, Julia Zuschnegg, Franziska Großschädl, Thomas E. Dorner, Regina Roller-Wirnsberger, Wolfgang Freidl

**Affiliations:** 1 Institute of Social Medicine and Epidemiology, Medical University of Graz, Graz, Austria; 2 Institute of Nursing Science, Medical University of Graz, Graz, Austria; 3 Academy for Ageing Research, Haus der Barmherzigkeit, Vienna, Austria; 4 Department of Internal Medicine, Medical University of Graz, Graz, Austria

**Keywords:** Long-term care, Last years of life, Age at death, Cause of death, End of life, Dementia, Parkinson’s disease, Cancer

## Abstract

Analyses of late-life disability based on survey data of the oldest old often suffer from non-representative samples due to selective participation and attrition. Here, we use register data on the Austrian long-term care allowance (ALTCA) as a proxy for late-life disability. In this retrospective mortality follow-back study, we analyze receipt of ALTCA, a universal cash benefit based on physician-assessed disability in activities of daily living during the last 10 years of life, among all decedents aged 65 years and over from 2020 in Austria (*n* = 76,781) and its association with sex, age at death, and underlying cause of death. We find that on average, ALTCA was received for 3.5 and 5.3 years in men and women. At 10 years before death, 10% of men and 25% of women received ALTCA, which increased to 56% and 77% at one year before death. Both the probability and duration of ALTCA increased with age at death and varied by cause of death: Those who died from cancer, myocardial infarction, and external causes of death were less likely to receive ALTCA and for shorter durations, while those who died from dementia, Parkinson’s disease, chronic heart disease, or chronic lung disease were more likely to receive it and longer so. Overall, our register-based estimates of the prevalence of late-life disability were higher than previous survey-based estimates. Policy-makers should be aware that costs of long-term care will rise as life expectancy rises and deaths from dementia and chronic heart disease will likely increase in the rapidly aging European societies.

**Supplementary Information:**

The online version contains supplementary material available at 10.1007/s10433-024-00823-z.

## Introduction

Many older adults experience a period of disability, that is, difficulty or dependence (Gill et al. 1998) in basic (Katz et al. 1963) (ADLs, e.g., eating, bathing, using the toilet or dressing) and instrumental (Lawton and Brody 1969) (IADLs, e.g., shopping, taking medication, cleaning), activities of daily living toward the end of life (Gill et al. 2010). Whether and when late-life disability sets in has profound implications for the affected older individuals and their families, as well as for societies at large who have to provide informal or formal (professional) long-term care (LTC) (OECD 2011) when older adults cannot perform everyday activities on their own. High-quality data on late-life disability among older adults, however, are not easy to obtain. Previous research relied on longitudinal survey data from later deceased older adults, providing information on self-reported ADLs during the last (Gill et al. 2010; Lunney et al. 2003), the last two (Smith et al. 2013), three (Guralnik et al. 1991; Liu et al. 2018), five (Stolz et al. 2021), ten (Stolz et al. 2019), twelve (Klijs et al. 2010), or even twenty (Chiu, et al. 2021a, b) years of life. These studies show that (1) disability is common during the last years of life, (2) its prevalence and severity increase toward death, and (3) the period of late-life disability often spans multiple years. In addition to higher disability levels in women, these studies also showed that disability prevalence and severity increased with age at death (Guralnik et al. 1991; Klijs et al. 2010; Liu et al. 2018; Smith et al. 2013) and varied by cause of death (Chiu et al. 2021a, b; Lunney et al. 2003; Stolz et al. 2021).

However, when it comes to older adults in general and the oldest old specifically, even high-quality longitudinal survey data often suffers from (small) non-representative samples due to restricted sampling frames as well as selective participation and attrition, which threats external validity (Chatfield et al. 2005; Drivsholm et al. 2006; Enzenbach et al. 2019; Gaertner et al. 2016; Hoeymans et al. 1998; Hunger et al. 2013; Kelfve et al. 2013; Kempen and van Sonderen 2002; Nummela et al. 2011; Wagner et al. 2019). A Dutch study (Hoeymans et al. 1998), for example, found that compared to respondents, non-respondents—who were later re-approached and successfully interviewed—were two times as likely to have had a stroke and more than three times as likely to be disabled with regard to ADLs and mobility. In other words, older adults with compromised health and disabilities often cannot and/or want not to participate in (repeated) survey studies, and if they do participate, they have a higher probability to drop out at some point before death due to health issues. These selection effects likely result in an underestimation of both the prevalence and the duration of late-life disability in previous work.

A potential alternative to survey studies to obtain population-representative information are individual-level register data based on administrative records (Thygesen and Ersbøll 2014), although these typically do not provide direct information on disability but instead document LTC service use (Forma et al. 2007; Hunger et al. 2013; Martikainen et al. 2012; Meinow et al. 2020). Using Swedish register data, Kelfve and colleagues (Kelfve et al. 2023), for example, recently reported that among LTC users, older women and men use residential or home care services on average 3.3 and 1.9 years at the end of their lives. Use of LTC services, however, is not an ideal proxy for late-life disability, as even in the case of Sweden where virtually all LTC is publicly funded, LTC service use is considerably higher among those older adults who live alone compared to those who have a cohabiting and care-providing spouse. Consequently, there are limits as to what can be learned about late-life disability by looking only at LTC service use.

Newly made accessible register data with individual linkage in Austria may help circumvent this problem. In contrast to Sweden and other Northern European countries with universalistic and service-oriented LTC regimes (OECD 2011), the main pillar of the Austrian LTC system is a tax-based cash benefit, the universal and strictly needs-based Austrian long-term care allowance (ALTCA, ‘Pflegegeld’), which is paid to all Austrian residents with a disability expected to last longer than six months (Österle 2013). For ALTCA, authorized physicians (first assessments and reassessments) and nurses (reassessments) use a standardized template during home visits to assess the level of disability based on the hours of care needed with ADLs and IADLs, which then determines the amount of the cash benefit. ALTCA is (1) well established and well known (only minor changes since its implementation in 1994), (2) reasonably fast (the duration between application, in-person assessment and decision usually takes about two months), (3) accessible (can be requested by the care-dependent older adult, informal care givers or professional care providers), (4) allows for re-assessment when the level of disability increases, (5) provides broad coverage (Ranci et al. 2019) (in 2020, 48% of Austrian residents aged 80 years and above received ALTCA), and (6) is substantial (in 2020, it ranged from 160€ per month at level 1 to 1719€ at level 7). Community-dwelling older adults can use the cash benefit discretionary; that is, they may use it to co-pay professional care services, give it to care-providing family members, or the allowance might just add to the household income. Older adults who receive ALTCA of level-3 or higher can be admitted to nursing homes, in which case ALTCA is subsequently largely transferred to the care home. Given these characteristics, we expect that receipt of ALTCA constitutes a useful proxy for the presence of late-life disability in the older population in Austria. In this retrospective mortality follow-back study, we use register-based information on the receipt of ALTCA to approximate both the probability and duration of disability during the last 10 years of life as well as the impact of sex, age at, and cause of death thereon.

## Methods

### Data

All register data were prepared by and accessed through the Austrian Micro Data Center (AMDC project N2B80) at Statistics Austria. According to the Austrian cause of death statistics, 78,802 older adults aged 65 years and above died in Austria in 2020. Valid data for ALTCA from integrated wage and income tax statistics and the covariates from labor market statistics were available for 76,781 (97.4%) decedents, which constitute the analytical sample (Supplementary Fig. 1).

### Variables

The primary outcome variable was the total number of months older adults received ALTCA during the last 10 years before their death, i.e., in the 120 months preceding their death in 2020. The secondary outcome is whether or not ALTCA was received during the last 10 years of life. Supplementary Table 1 summarizes which ADLs and IADLs are considered during the at-home assessments conducted by authorized physicians and nurses. Importantly, the assessment focuses on the ability or inability of older adults to perform tasks, not whether they actually engage in them. For example, residents in long-term care facilities are often provided with meals and so do not need to prepare these themselves. Nonetheless, if the older adult in question is judged by the authorized physician or nurse to not be *capable* of preparing meals (including doing the dishes) given their physical, cognitive, or mental status, this will be recognized as a care need of up to one hour per day (Supplementary Table 1). In addition to these specified time needs in ADLs and IADLs, there is a flat-rate premium in case of dementia and defined minimum allowance levels for certain diagnoses, for example, paraplegia or blindness (about 5% of cases). The total hours of assessed care need with ADLs and IADLs subsequently determine the level of ALTCA, ranging from 1 to 7 (Supplementary Table 2). As an entry criterion to obtain ALTCA (level 1), a total of at least 2.2 recognized daily hours of care need is required. Unfortunately, information on the level of ALTCA cannot be fully reconstructed from wage and income statistics, and therefore, available information is restricted to the number of months older adults received ALTCA before death.

Information on age at death (in years) and underlying cause of death came from cause of death statistics which follows the 10th edition of the International Classification of Diseases (ICD-10). We used two alternative classifications for cause of death: major ICD-10 categories (taxonomy-based) and frequent single diagnoses (frequency-based). For the taxonomy-based classification, ICD-10 categories included infectious/parasitic (ICD-10: A00-B99), neoplasms (C00-D48), endocrine/nutritional/metabolic (E00-E90), mental/behavioral (F00-F99), nervous system (G00-G99), circulatory system (I00-I99), respiratory system (J00-J99), digestive system (K00-K93), genitourinary system (N00-N99), non-classified (R00-R99), or external causes (V01-Y98). ICD-10 categories with less than 500 deaths (blood/immune system (D50-D89), skin (L00-L99), musculoskeletal system (M00-M99), and congenital (Q00-Q99)) were collapsed to ‘other.’ For the frequency-based classification, we included all single underlying cause of death diagnoses that accounted for more than 1% of the total population, stratified by sex. All causes of death with less than 1% prevalence were again collapsed to ‘other’ causes of death.

Covariates included sex (male, female), level of completed education (ISCED-2011: primary or lower secondary, vocational school, upper secondary, post-secondary, tertiary), and marital status at time of death (married, single, divorced, widowed).

### Statistical analysis

First, we described the characteristics of the study population. Second, we used a statistical model to isolate the effect of age at death and cause of death on receipt of ALTCA during the last decade of life from each other. As a robustness check (supplementary material), we also adjusted results for sociodemographic background variables (education, marital status), which might influence both disability in old age as well as whether and when a request for ALTCA is filed. All analyses were stratified by sex. The main outcome, the number of months of ALTCA a person obtained is an integer variable with a strict lower (0, i.e., none) and upper bound (120, i.e., throughout the last 10 years of life). The outcome variable showed a strong bimodal distribution; hence, a linear regression model cannot not provide an adequate reproduction of these data. Unfortunately, generalized linear regression models often used for count data such as the Poisson or the negative binomial model are also ill-equipped to integrate the substantial number of extreme values (0 and 120 months). To model the months of ALTCA, we instead used the zero-and-one-inflated beta regression model (Ospina and Ferrari 2012), a recently developed and more appropriate statistical approach for this kind of mixed categorical and continuous data. This model uses a mixture of the beta-distribution to represent the continuous component of the data (1–119 months) expressed as a proportion (1/120 = 0.008, 119/120 = 0.992) and the Bernoulli distribution for whether the outcome is an extreme value (0 = 0,120 = 1), and if so, whether it is the upper extreme. An advantage of this approach is that the three sub-models are independent from each other, i.e., the coefficients may vary between the continuous and the categorical model parts so that differential processes are accounted for. This, however, renders the model output complex, and to obtain a single set of coefficients, we estimated the predicted number of months based on the overall model for the variable in question holding the rest constant at their median or mode. For the secondary outcome, that is, the probability that ALTCA was *not* received before death, we used a standard logistic regression model. For more details about the models, see the R-code (https://osf.io/3acun/). All models were estimated with R-package ‘*brms*’ (Bürkner 2017) (2.20-4), a front-end for Stan (2.32-2) in R (4.1.3).

## Results

From the 76,781 decedents, 52.7% were women and 47.3% were men. In this population of decedents born between 1911 and 1955, 45.5% had completed primary or lower secondary school, 39.2% vocational school, 2.8% upper secondary, 8.5% post-secondary, and 4.0% tertiary education. Compared to women, men had more often completed post-secondary or tertiary education (Table 1). At the time of death, 38.6% of the older decedents were married, 7.6% single, 9.3% divorced, and 44.5% widowed. Women were considerably more likely to be widowed (62.5%) than men (24.6%).

**Table 1:** Sample characteristics

	Total (*n* = 76,781)	Men (*n* = 36,330)	Women (*n* = 40,451)
	*N* (%)	*N* (%)	*N* (%)
Education			
Primary/lower secondary	34,939 (45.5)	10,469 (28.8)	24,470 (60.5)
Vocational school	30,083 (39.2)	18,254 (50.2)	11,829 (29.2)
Upper secondary	2178 (2.8)	1085 (3.0)	1093 (2.7)
Post-secondary	6490 (8.5)	4278 (11.8)	2212 (5.5)
Tertiary	3091 (4.0)	2244 (6.2)	847 (2.1)
Marital status			
Married	29,626 (38.6)	21,407 (58.9)	8129 (20.3)
Single	5800 (7.6)	2665 (7.3)	3135 (7.8)
Divorced	7168 (9.3)	3333 (9.2)	3835 (9.5)
Widowed	34,187 (44.5)	8925 (24.6)	25,262 (62.2)
Cause of death (ICD-10 category, taxonomic approach)			
A00-B00	757 (1.0)	340 (0.9)	417 (1.0)
C00-D49	16,610 (21.6)	9014 (24.8)	7596 (18.8)
E00-E89	3251 (4.3)	1530 (4.2)	1721 (4.3)
F00-F99	2775 (3.6)	1045 (2.9)	1730 (4.3)
G00-G99	3001 (3.9)	1319 (3.6)	1682 (4.2)
I00-I99	29,709 (38.7)	12,843 (35.4)	16,866 (41.7)
J00-J99	4319 (5.6)	2289 (6.3)	2030 (5.0)
K00-K95	2262 (2.9)	1079 (2.9)	1183 (2.9)
N00-N99	2189 (2.9)	846 (2.3)	1343 (3.3)
R00-R99	2258 (2.9)	1077 (3.0)	1181 (2.9)
U00-U85	5946 (7.7)	3074 (8.5)	2872 (7.1)
V00-Y99	3118 (4.6)	1664 (4.6)	1454 (3.6)
Other	586 (0.7)	210 (0.6)	376 (0.9)
	Median (IQR)	Median (IQR)	Median (IQR)
Age at death: median (IQR)	85.5 (12.6)	81.5 (12.4)	87.1 (12.0)

*ICD-10* international classification of diseases, 10th edition. *IQR* interquartile range. *A00-B00* infectious/parasitic diseases, *C00-D48* neoplasms, *E00-E90* endocrine/nutritional/metabolic, *F00-F99* mental/behavioral, *G00-G99* nervous system, *I00-I99* circulatory system, *J00-J99* respiratory system, *K00-K93* digestive system, *N00-N99* genitourinary system, *R00-R99* non-classified, *U00-U85* provisional assignment of new diseases of uncertain etiology or emergency use, *V01-Y98* external causes of death

**Fig. 1:**
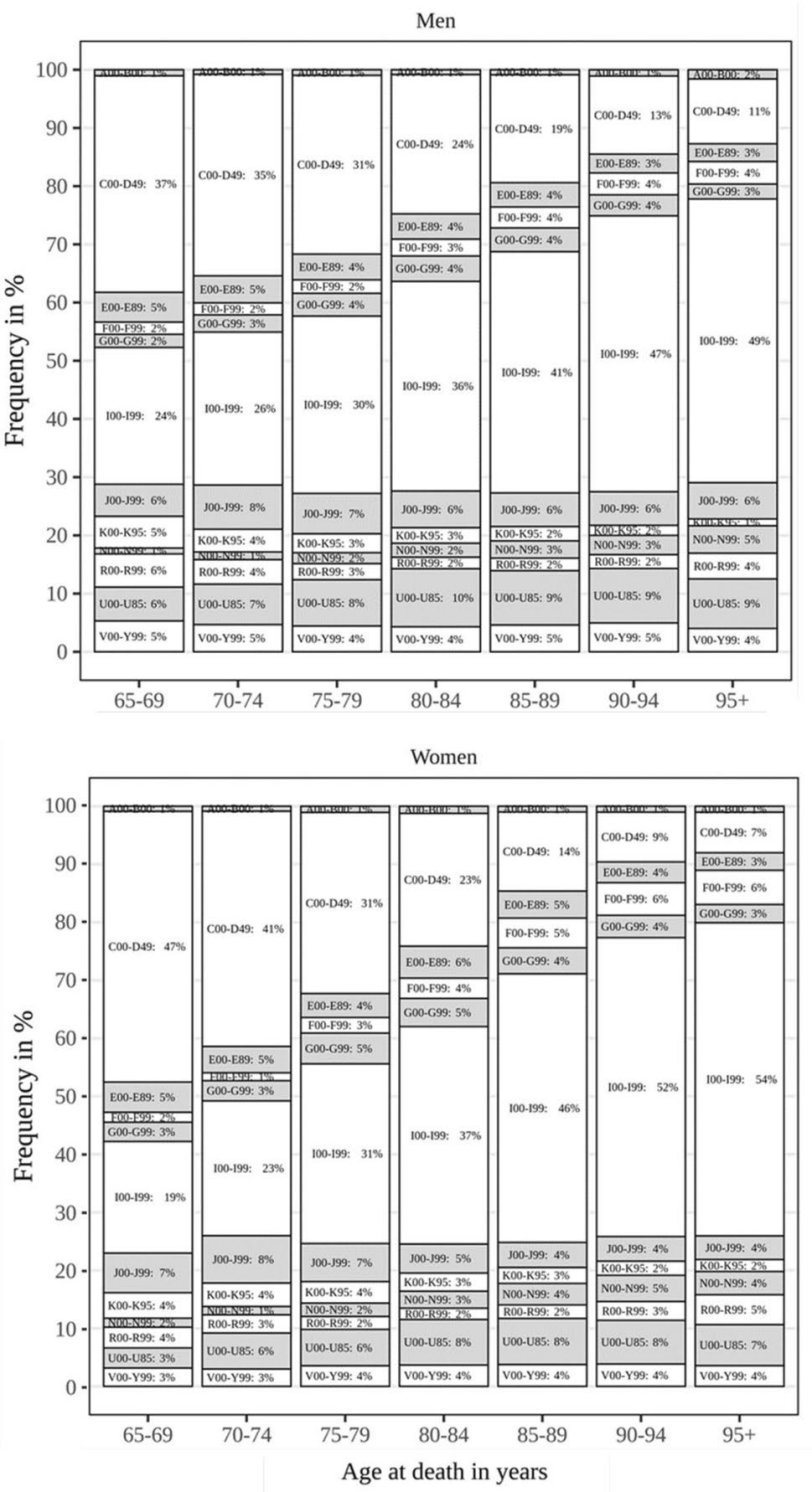
Cause of death categories by age at death and sex. Total number of decedents aged 65 years and above = 76,781, men = 36,330, women = 40,451. ALTCA = Austrian long-term care allowance, A00-B00 = infectious/parasitic diseases, C00-D48 = neoplasms, E00-E90 = endocrine/nutritional/metabolic, F00-F99 = mental/behavioral, G00-G99 = nervous system, I00-I99 = circulatory system, J00-J99 = respiratory system, K00-K93 = digestive system, N00-N99 = genitourinary system, R00-R99 = non-classified, U00-U95 = provisional assignment of new diseases of uncertain etiology or emergency use, V00-Y99 = external causes of death

Median age at death was 81.5 (IQR = 12.4) years in men (range = 65–107), and 87.1 (IQR = 12.0) years in women (range = 65–110). The two most important categories of underlying causes of death were cardiovascular diseases (ICD-10: I00-I99; CVD) amounting to 38.7% and cancers (C00-D49) with 21.6%, with women being more likely to die from CVD (41.7% vs. 35.4%) than men, and less likely to die from cancer (18.8% vs. 24.8%). The five most common single causes of death among older women in 2020 were atherosclerotic heart disease (ICD-10: I25.1 = 7.5%), COVID-19 (ICD-10: U071 = 6.7%), unspecified dementia (ICD-10: F03 = 3.7%), breast cancer (ICD-10: C50.9 = 3.0%), and acute myocardial infarction (ICD-10: I21.9 = 2.9%). The five most prevalent single causes of death among older men were COVID-19 (ICD-10: U071 = 8.2%), atherosclerotic heart disease (ICD-10: I25.1 = 7.5%), acute myocardial infarction (ICD-10: I21.9 = 4.2%), lung cancer (ICD-10: C34.9 = 4.1%), and prostate cancer (ICD-10: C61.0 = 3.6%). The percentage of deaths due to cancer decreased with age at death, while the percentage of deaths from CVD as well as from diseases of the mental and nervous as well as the genitourinary system and COVID-19 increased with age at death (Fig. 1).

The number of months for which ALTCA was obtained during the last decade of life showed a sex-specific, bimodal distribution (Supplementary Fig. 2): 30.5% of men and 13.0% of women had not obtained any ALTCA before death (= 0 months), and about 60% in both sexes received it for 1–119 months, while 10.3% of men and 25.4% of women had received it already 10 years before death, i.e., throughout the last decade of life. Five years before death, 26.7% (men) and 51.3% (women) received ALTCA, which further increased to 55.8% and 78.6% 1 year before death, and finally to 69.5% and 87.0% 1 month before death (Table 2).

**Table 2:** Proportion of ATLCA recipients across time before death

Time point before death	Total (%)	Men (%)	Women (%)
10 years	18.3	10.3	25.4
9 years	21.3	12.4	29.3
8 years	25.3	14.8	34.7
7 years	29.2	17.9	39.4
6 years	34.2	21.9	45.2
5 years	39.7	26.7	51.3
4 years	45.8	32.1	58.1
3 years	52.2	38.9	64.2
2 years	59.5	46.5	71.1
1 year	67.8	55.8	78.6
6 months	73.3	62.5	82.9
1 month	78.7	69.5	87.0

Total number of decedents aged 65 years and above = 76,781, men = 36,330, women = 40,451. ALTCA = Austrian long-term care allowance

The estimated average number of months for which ALTCA was obtained during the last decade of life (for a person of median age and with the mode cause of death, i.e., CVD) was 42.3 (95%-CI = 41.5, 43.1) months for older men and 63.9 (95%-CI = 63.1, 64.7) months for older women. This amounts to 3.5 years for men and 5.3 years for women, i.e., a 1.8-year difference (+ 51%).

**Fig. 2:**
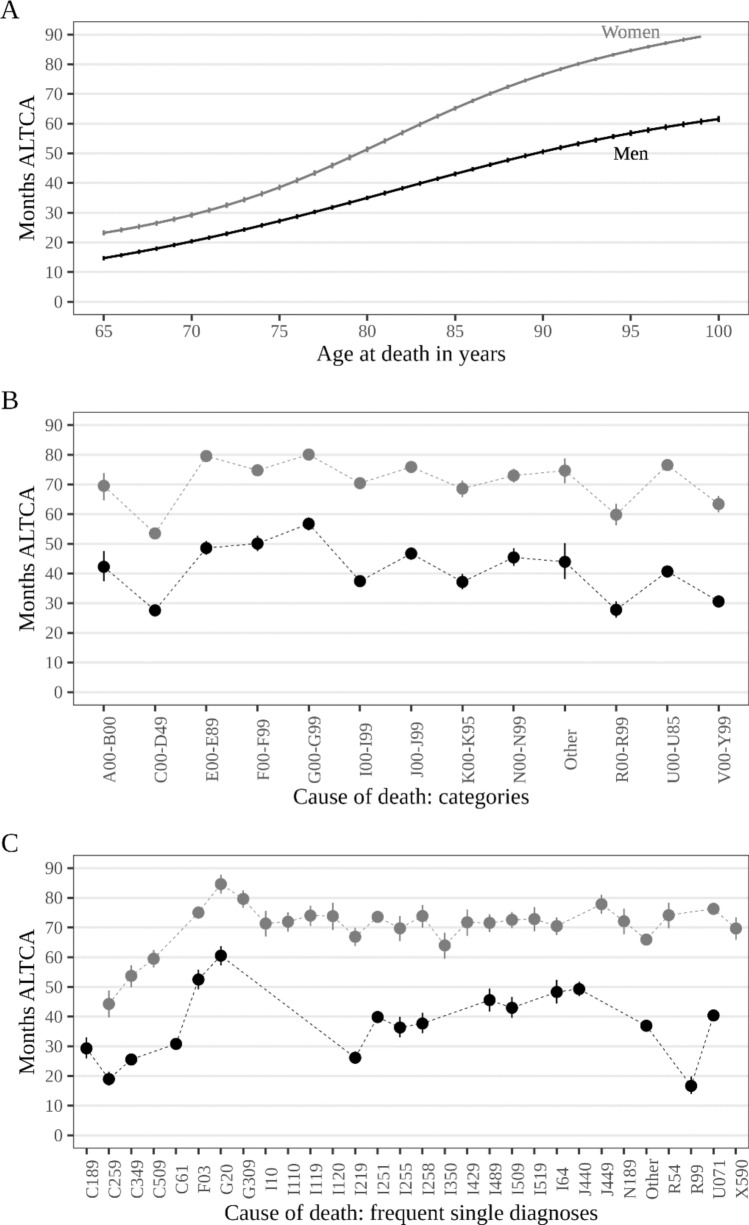
Estimated months of receipt of ALTCA before death by sex, age, and cause of death. Estimates based on zero–one-inflated beta regression models based on 76,781 observations. Lines (panel **A**) and points (panels **B**, **C**) refer to point estimates, and vertical lines (panel **A**–**C**) show 95% credible intervals. Estimates refer to older adults who died from cardiovascular diseases (ICD-10: I00-I99) (Panel **A**) and who died at median age at death (Panels **B**, **C**). (ALTCA = Austrian long-term care allowance, A00-B00 = infectious/parasitic diseases, C00-D48 = neoplasms, E00-E90 = endocrine/nutritional/metabolic, F00-F99 = mental/behavioral, G00-G99 = nervous system, I00-I99 = circulatory system, J00-J99 = respiratory system, K00-K93 = digestive system, N00-N99 = genitourinary system, R00-R99 = non-classified, U00-U95 = provisional assignment of new diseases of uncertain etiology or emergency use, V00-Y99 = external causes of death. C189 = colon cancer, C259 = pancreas cancer, C349 = lung cancer, C50.9 = breast cancer, C61 = prostate cancer, F03 = unspecified dementia, G20 = Parkinson’s disease, G309 = Alzheimer’s disease, I10 = hypertension, hypertensive heart disease with heart failure, I119 = hypertensive heart disease without heart failure, I120 = hypertensive renal disease with renal failure, I21.9 = acute myocardial infarction, I251 = atherosclerotic heart disease, I255 = ischemic cardiomyopathy, I258 = other forms of ischemic heart disease, I350 = aortic stenosis, I429 = unspecified cardiomyopathy, I489 = atrial fibrillation, I509 = unspecified heart failure, I64 = stroke, J440 = chronic obstructive pulmonary disease with acute lower respiratory infection, J449 = unspecified chronic obstructive pulmonary disease, N18.9 = chronic kidney disease, R54 = senility, R99 = other ill-defined or unspecific cause of death, U071 = COVID-19, virus identified, X590 = fracture with unknown or unspecified circumstance)

Figure 2 shows that the duration of ALTCA increased substantially with the age at death (panel A), that is, those who died with higher age received ALTCA for longer: at age 65, this was a bit over one-year (men) and two years (women) and increased to three (men) and six (women) years at the median age at death. This relative sex-difference diverged further among those who died at age 90 and above.

Panel B of Fig. 2 shows that for both, men and women, some groups of underlying causes of death were associated with a shorter duration of ALTCA, notably death due to cancer (ICD-10: C00-D49), and unclassified (ICD-10: R00-R99) or external causes of death (ICD-10: V01-Y98), while death from endocrine/nutritional/metabolic (ICD-10: E00-E90), mental/behavioral (ICD-10: F00-F99) diseases, and particularly diseases of the nervous system (ICD-10: G00-G99) were associated with a longer duration of ALTCA. The results for frequent single causes of death (Panel C) also show that among both men and women, cancer deaths were associated with fewer months of ALTCA, particularly pancreatic cancer (ICD-10: C25.9). For men, acute myocardial infarction (ICD-10: I21.9) and unclassified deaths (ICD-10: R99) were also associated with a considerably shorter period of ALTCA. The longest duration of ALTCA in both men and women among the single diagnoses was found for unspecified dementia (ICD-10: F03), Alzheimer’s disease (ICD-10: G30.9), and Parkinson’s disease (ICD-10: G20), followed by death due to chronic obstructive lung disease (ICD-10: J44.9) and chronic heart diseases (ICD-10: I11.9, I20.0, I25.5, I25.8) among women, and stroke (ICD-10: I64) and chronic obstructive lung disease (ICD-10: J44.0) among men. The difference between causes of death associated with the shortest and the longest duration of ALTCA before death was substantial and amounted up to 2–3 years. Notably, older adults who died from COVID-19 (ICD-10: U071) received ALTCA for an average (men) or even above-average (women) duration. For more detailed results, see Supplementary Tables 3, 4. Adjusting for marital status and education level did not change these results substantively (Supplementary Fig. 3).

**Fig. 3:**
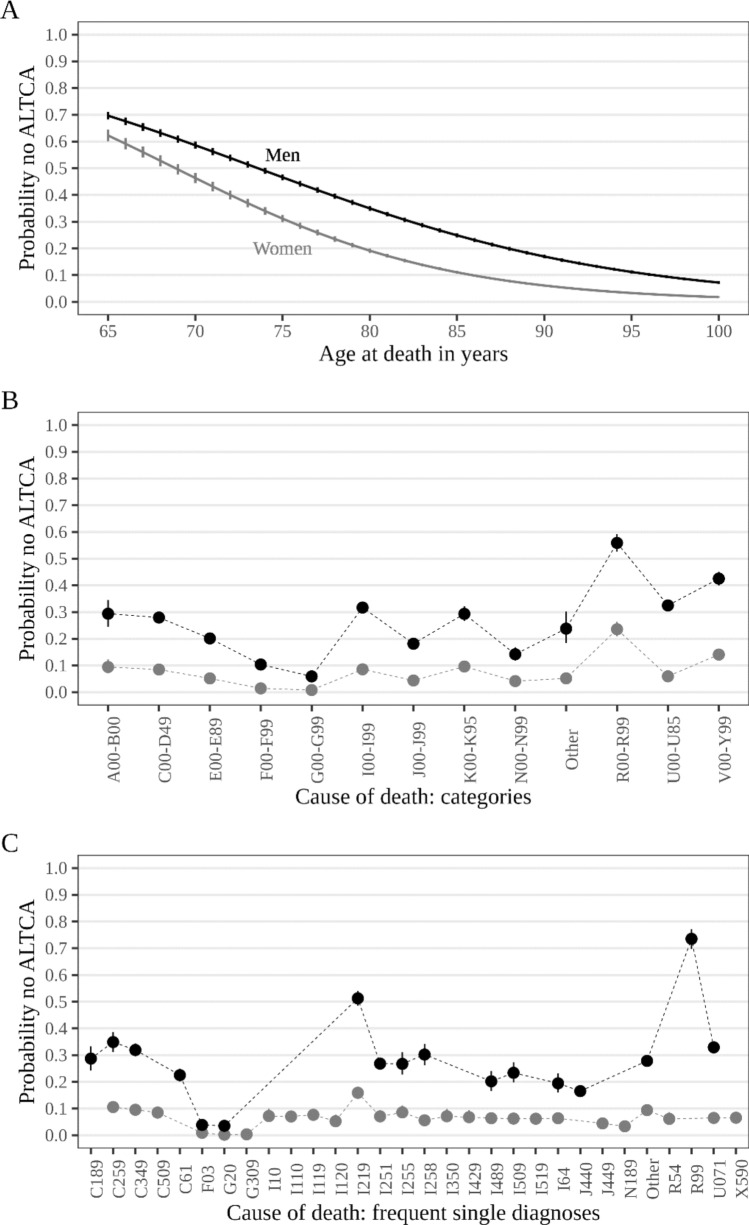
Probability to not receive ALTCA before death by sex, age, and cause of death. Estimates based on logistic regression models based on 76,781 observations. Lines (panel **A**) and points (panels **B**, **C**) refer to point estimates, and vertical lines (panel **A**–**C**) show 95% credible intervals. Estimates refer to older adults who died from cardiovascular diseases (ICD-10: I00-I99) (Panel **A**) and who died at median age (Panels **B**, **C**). ALTCA = Austrian long-term care allowance, A00-B00 = infectious/parasitic diseases, C00-D48 = neoplasms, E00-E90 = endocrine/nutritional/metabolic, F00-F99 = mental/behavioral, G00-G99 = nervous system, I00-I99 = circulatory system, J00-J99 = respiratory system, K00-K93 = digestive system, N00-N99 = genitourinary system, R00-R99 = non-classified, U00-U95 = provisional assignment of new diseases of uncertain etiology or emergency use, V00-Y99 = external causes of death. C189 = colon cancer, C259 = pancreas cancer, C349 = lung cancer, C50.9 = breast cancer, C61 = prostate cancer, F03 = unspecified dementia, G20 = Parkinson’s disease, G309 = Alzheimer’s disease, I10 = hypertension, hypertensive heart disease with heart failure, I119 = hypertensive heart disease without heart failure, I120 = hypertensive renal disease with renal failure, I21.9 = acute myocardial infarction, I251 = atherosclerotic heart disease, I255 = ischemic cardiomyopathy, I258 = other forms of ischemic heart disease, I350 = aortic stenosis, I429 = unspecified cardiomyopathy, I489 = atrial fibrillation, I509 = unspecified heart failure, I64 = stroke, J440 = chronic obstructive pulmonary disease with acute lower respiratory infection, J449 = unspecified chronic obstructive pulmonary disease, N18.9 = chronic kidney disease, R54 = senility, R99 = other ill-defined or unspecific cause of death, U071 = COVID-19, virus identified, X590 = fracture with unknown or unspecified circumstance

Finally, the results from the logistic regression models provide a similar overall picture (Fig. 3): The probability to die *without* receiving ALTCA (panel A) shrinks drastically with increasing age at death: from between 60 and 70% for men and women at an age at death of 65 years to 27% for men and 11% for women who died at the age of 85. For those who died aged 95, the probability to die without receiving ALTCA before death was only 11% in men and 3% in women. Holding age at death constant (at the median), we see that the probability to not receive ALTCA when subsequently dying from the two most common major causes of death, CVD and cancer, was about 30% in men and 10% in women. Compared to women, there was more variance across causes of death in men (panel C). Acute myocardial infarction (ICD-10: I21.9) again stood out, being associated with a probability of over 50% in men to die without previously receiving ALTCA. Interestingly, death from COVID-19 (ICD-10: U071) was also associated with a considerable probability to die without receiving ALTCA among men (33%), but not among women (6%). In contrast, the chance to die without having received ALTCA in case of dementia or Parkinson’s disease was minimal (< 5%) for both sexes. For more detailed results, see Supplementary Tables 5, 6. Adjusting for marital status and education level again did not change these results substantively (Supplementary Fig. 4).

## Discussion

In this study, we found that only a small (13%) minority of deceased older women and a significant minority of older men (31%) had *not* received ALTCA before death; that is, a large majority of older adults in Austria experienced a period of physician-assessed and officially recognized disability in ADLs and IADLs before death. The prevalence of late-life disability as approximated by ALTCA increased strongly during the last 10 years of life, and the estimated average duration was 3.5 years in older men and 5.3 years in older women. Both the probability and duration of receiving ALTCA increased strongly with age at death and varied by the underlying cause of death. Older adults who died from cancer, myocardial infarction, or external causes of death received it for fewer months and had a higher probability to die without it, while those who died from dementia, Parkinson’s disease, or chronic heart or lung disease were more likely to receive ALTCA before death and longer so.

In our study, receipt of ALTCA increased substantially during the last years of life and was high during the very last years, which is compatible with previous results on late-life disability (Chiu et al. 2021a, b; Gill et al. 2010; Guralnik et al. 1991; Liu et al. 2018; Lunney et al. 2003; Smith et al. 2013; Stolz et al. 2021). However, compared to disability prevalence estimates based on survey data, our register-based estimates were substantially higher. Gill et al. (2010), for example, reported that among 383 deceased participants from a longitudinal study of 754 community-dwelling insurance plan members, less than half needed help in one or more ADLs one year before death. Based on a larger sample of US-wide decedents, Smith et al. (2013) estimated that 36% were ADL-dependent at one year before death. Finally, a study from Taiwan (Chiu et al. 2021a, b) reported a 46% prevalence of ADL disability one year before death. Approximated via the receipt of ALTCA, our register-based estimates of at least 2.2 or more hours of daily care need with ADLs and IADLs during the last 10 years of life were almost twice as high. This discrepancy could be due to the fact that we were able to leverage complete population data and hence avoid restrictive sampling frames that often exclude institutionalized older adults (Kelfve et al. 2013) as well as selective participation and attrition effects (Chatfield et al. 2005; Drivsholm et al. 2006; Enzenbach et al. 2019; Gaertner et al. 2016; Hoeymans et al. 1998; Hunger et al. 2013; Kelfve et al. 2013; Kempen and van Sonderen 2002; Nummela et al. 2011; Wagner et al. 2019) that likely down-bias existing survey-based estimates. However, other reasons might also account for this gap as comparability across studies is limited due to differences in definition and measurement of outcomes, inclusion criteria including age ranges, and national contexts more generally. Austria, for example, ranks below average within Europe with regard to estimated healthy life expectancy (Welsh et al. 2021) so that we would expect to find a somewhat lower prevalence of late-life disability in Northern European countries in comparison. This fits with our estimates of receipt of ALTCA being higher compared to Swedish (Kelfve et al. 2023) register-based estimates of LTC service use. Again, however, direct comparisons are difficult as the Swedish study referred to residential and home care service use and included only those who entered professional long-term care.

A higher age at death was associated with a higher probability and longer duration of receipt of ALTCA before death in our study, which is compatible with previous results on late-life disability (Guralnik et al. 1991; Klijs et al. 2010; Liu et al. 2018; Smith et al. 2013). Our estimates imply a sharp increase of ADL and IADL disability prevalence with age at death, affecting > 90% among the oldest old. Our results with regard to the impact of age at death are also compatible with a previous study (Forma et al. 2007) based on Finnish register data, where institutional or 24 h LTC service use during the last two years of life was shown to increase from 26% (men) and 37% (women) among those who died aged 70–79 years to 64% and 78% among those aged ≥ 90 years at death. Again, our results imply an even higher effect and were robust to adjustments for sociodemographic background variables (marital status and level of education). In sum, our findings imply a strong gradient: The older individuals die, the more likely and the longer they can expect to need help with ADLs and IADLs before death. A rationale for this finding is that with increasing age, older adults tend to accumulate more and more chronic diseases and health conditions (Rockwood and Mitnitski 2007) which result in impairments such as memory problems, muscle weakness, dizziness, fatigue, or pain that limit the ability to perform physical and mental actions which result in disability (Verbrugge and Jette 1994), first in IADLs and then ADLs (Stolz et al. 2021).

Another clear finding from our study was that women lived not only longer but also received ALTCA for longer, which echoes the known gender-gap in disability (Crimmins et al. 2011) that has been attributed to more prevalent non-lethal chronic conditions such as arthritis, but also lower functioning already in midlife, lower muscle strength in old age as well as more sedentary behavior in women compared to men (Crimmins et al. 2011; Gill et al. 2013; Leveille et al. 2000; Nusselder et al. 2019).

In our study, we also found considerable differences in ALTCA, our register-based proxy of late-life disability, during the last 10 years of life with regard to the underlying cause of death. Previous studies (Chiu et al. 2021a, b; Lunney et al. 2003; Stolz et al. 2021) have reported distinct disability trajectories: Older adults who die from cancer have been reported to have few disabilities up until several months before death, when a steep terminal decline sets in. In contrast, older adults who died from chronic heart or lung disease and dementia experienced a longer period of higher levels of late-life disability that increased more gradually. Our results are compatible with these findings. A longer nursing home care use during the last years of life for those dying from dementia compared to other causes of death has also been reported based on register data from Finland (Martikainen et al. 2012). In contrast to these studies, our study lacked a (longitudinal) measure of disability severity and hence provides no evidence on the timing of (terminal) declines in functioning (Landré et al. 2021). Of note, among older men, we also found that ill-defined or unknown causes of death (ICD-10: R99) were particularly associated with a high probability to die without previously receiving ALTCA. Finally, as our study referred to decedents from 2020, the first year of the COVID-19 pandemic, we found that COVID-deaths were among the most frequent single causes of death. Up to this point, little has been known about the late-life disability profile among older adults who died from COVID-19. Here, we found that COVID-19 deaths received ALTCA for an average or even above-average duration, which points to death through COVID-19 among often already care-dependent older adults in the community and nursing homes (Levin et al. 2022; Weaver 2024). At the same time, there was indeed a substantial chance for a ‘sudden death’ (Lunney et al. 2003), that is, death without preceding ALTCA, among older men which fits with the generally higher mortality risk for older men compared to women by the infectious disease that is COVID-19 (Dessie and Zewotir 2021).

Next to its strengths, that is, individual-level register data for all decedents in Austria and linkage with information on age and cause of death, which has only become possible recently, there are also several noteworthy limitations to this study. First, and most importantly, we used receipt of ALTCA as a proxy variable for late-life disability. While ALTCA is well established, accessible, and substantial—all of which should incentivize most care-dependent older adults, as well as their relatives and professional care providers in Austria to apply for it as soon as substantial care needs are recognized—there are also reasons why disabled older adults might not take up the cash benefit, specifically lack of knowledge, language deficits, and individual cost–benefit considerations (Pennerstorfer and Österle 2023). Given the large gender-gap between older men and women in our results, it could be, for example, that older men are not only less inclined to report disabilities (Auais et al. 2019) but also to not apply for a cash benefit that requires an formal assessment of their inabilities—particularly in case affluent older individuals. On the other hand, it has been found (Merrill et al. 1997) that disability reporting is generally accurate for both older men and women, and higher reported disability among the latter likely reflects factual differences. Another limitation of our study is that more than 2.2 h of daily care need in ADLs and IADLs are required to qualify for ALTCA. Taken together, a selective uptake of the cash benefit and the minimum requirement could lead to an underestimation of the true prevalence of late-life disability due to our use of ALTCA as a proxy variable. However, given the high proportion of ALTCA recipients and the confirmation of expected associations with regard to sex, age at and cause of death in our study, as well as the standardized disability assessment through physicians and nurses, and the (almost) full-population coverage, we think that on balance, the receipt of ALTCA is an adequate register-based alternative to surveys for studying late-life disability. Another limitation is that currently, it is not possible to distinguish the degree of disability in ALTCA, which constitutes an advantage of available survey over register data. Also, for a small percentage of older decedents (2.6%) administrative data was not available, and our study included only decedents of a single year (2020), which was also the first year of the COVID-19 pandemic. The substantial number of COVID-19 deaths might also have changed pre-pandemic late-life disability patterns, although our results imply only a modest effect. A final important limitation stems from the principal difficulty of determining single causes of death in the presence of multiple comorbidities, which are common among older adults and increase with age (Grundy and Stuchbury 2022; Tinetti et al. 2012). Recently, new approaches of analyzing multimorbidity based on multiple-cause of death statistics have been suggested (Grippo et al. 2020; Grundy and Stuchbury 2022), which hold promise for future studies in this regard.

The implications of our results make for a somewhat dire reading. First, older adults in Austria, and presumably also other European countries, should be aware that they are likely to experience a multi-year period of disability before death where they will have to rely upon others for everyday activities of life. One in four older women can even expect to need care with at least some ADLs and IADLs *throughout* their last decade of life. Older adults and their family members should know this and plan accordingly. With regard to policy, our findings imply that late-life disability is an issue that directly or indirectly affects large parts of the population. Given the ageing of the large baby boomer cohort and the fact that older adults die at an ever-increasing age—and hence may also be more likely to die from (or with) dementia or chronic heart disease which are associated with longer periods of late-life disability as approximated by ALTCA—the already substantial costs for LTC will likely rise in the foreseeable future. Efforts to recruit and upkeep the professional LTC workforce to supplement the decreasing number of familial care givers will be vital to ensure that older adults care needs are met in the years ahead. Interventions reducing late-life disability, for example, by means of physical activity and exercise (Tak et al. 2013), may be less effective in the oldest old when disability is often already advanced. Therefore, prevention strategies should also aim to increase functioning in earlier life periods, that is, in late midlife and early old age to increase physical (and cognitive) reserves, so that older adults might be able to care for themselves and live independently for longer during their last decade of life.

## Conclusion

Most older adults in Austria, particularly older women, received ALTCA for a multi-year period; that is, they experienced prolonged late-life disability before their death, which increased strongly with age at death and varied across causes of death. Our register-based population-wide estimates of the prevalence of late-life disability as approximated by receipt of the physician-assessed ALTCA were higher than previous self-reported survey-based estimates, which points toward an underestimation due to selection bias in surveys. Older adults in Austria, and presumably also other European countries, should be aware of a lengthy period of late-life disability and policy-makers should be aware that costs of LTC will likely increase as longer life expectancy and deaths from dementia, and Parkinson’s disease and chronic heart disease will increase in the rapidly aging societies of Europe.

## Supplementary Information

Below is the link to the electronic supplementary material.

Supplementary file1 (DOCX 676 kb)

## Data Availability

The R-code used for data preparation and analysis is available online (https://osf.io/3acun/). The linked individual-level register data can be accessed via the Austrian Micro Data Center (AMDC project N2B80) but requires an accreditation of the scientific host institution and a formal request from researchers. Note that a fee applies for data preparation and provision of data access by the AMDC. For more information, please contact the AMDC (amdc@statistic.gv.at).
