# Supplementary material for: Disability during the last ten years of life: evidence from a register-based study in Austria
Source: Eur J Ageing. 2024 Sep 28;21(1):28. doi: 10.1007/s10433-024-00823-z (PMC11438747; doi:10.1007/s10433-024-00823-z)
Supplement: Supplementary file 1 — Supplementary file1 (DOCX 676 kb) [file 10433_2024_823_MOESM1_ESM.docx]

**Supplementary Material**

Supplementary Figure 1: Flowchart of study population

Supplementary Table 1: Basic and instrumental activities of daily living considered for ALTCA

Supplementary Table 2: Requirement and amount of ALTCA by level

Supplementary Figure 2: Distribution of the number of months of ALTCA during the last 10 years of life by sex

Supplementary Table 3: Estimated duration of ALTCA in months for men and women by cause of death (taxonomic approach)

Supplementary Table 4: Estimated duration of ALTCA in months for men and women by cause of death (frequency-based approach)

Supplementary Table 5: Estimated probability to not receive ALTCA for men and women by cause of death (taxonomic approach)

Supplementary Table 6: Estimated probability to not receive ALTCA for men and women by cause of death (frequency-based approach)

Supplementary Figure 1: Flowchart of study population


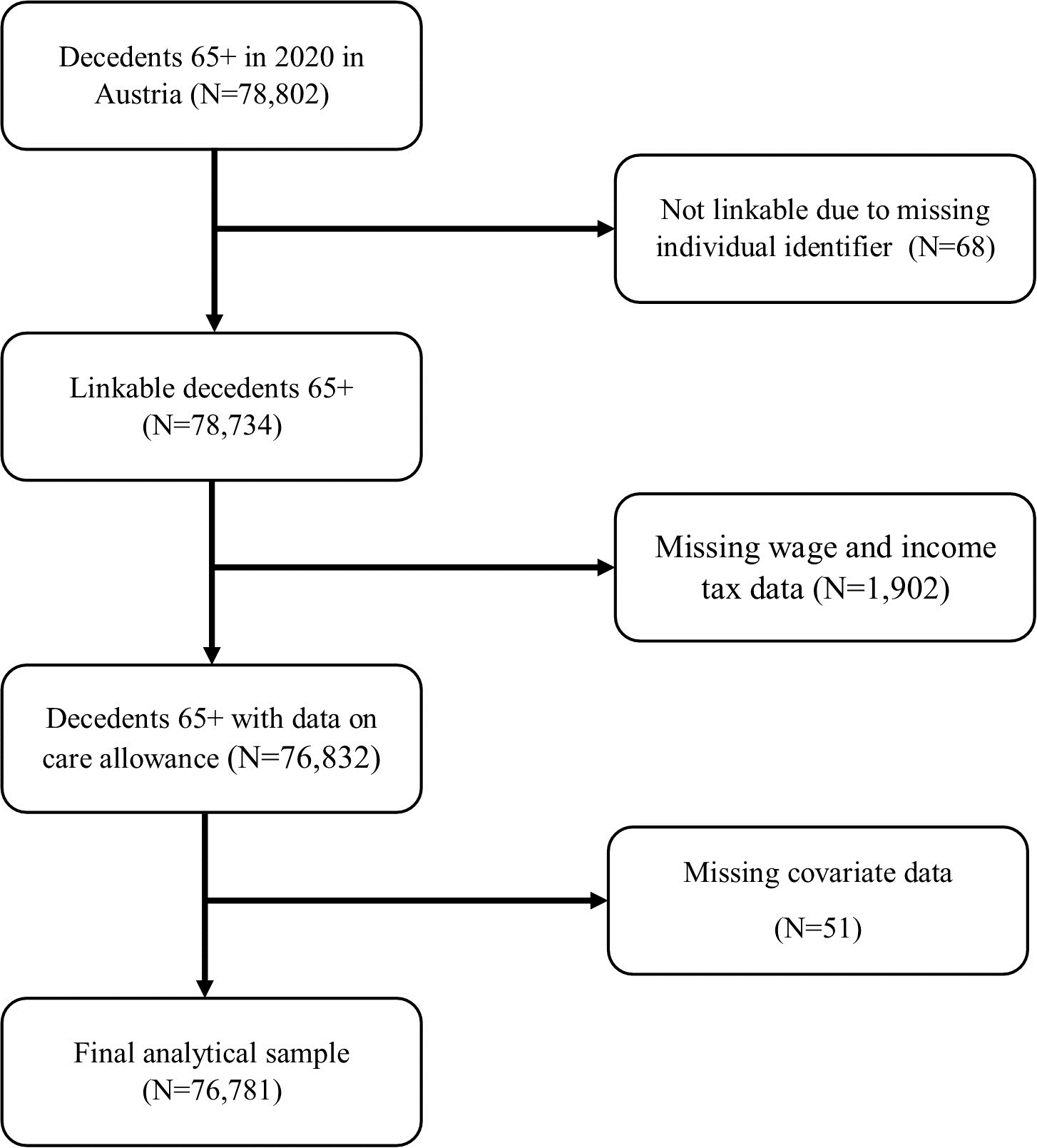


Supplementary Table 1: Basic (1) and instrumental (2) activities of daily living considered for ALTCA

| Activities of daily living | Maximum daily care need |
| --- | --- |
| (1) Daily body care (washing of face, hands, upper and lower body; tooth brushing, dress the hair, shaving) | 2.5 h |
| (1) Preparing meals (meals, drinks, washing dishes) | 3 h |
| (1) Eating | 3 h |
| (1) Using toilet (get into toilet, undress, use toilet, dress, washing hands) | 3 h |
| (1) Dressing (includes selection of clothing) | 2 h |
| (1) Cleaning in case of incontinence (change of diapers, cleaning lower body, undressing/dressing) | 2 h |
| (1) Mobility assistance within house | 1.5 h |
| (2) Assistance with cooking | 1 h |
| (2) Shopping groceries and medication | 1 h |
| (2) Cleaning of living space | 1 h |
| (2) Washing of underwear and bedclothes | 1 h |
| (2) Mobility assistance outside house | 1 h |
| (2) Medication (preparation and taking) | 0.1-0.17 h |
| Hardship allowance (flat rate) in case of dementia, strong cognitive limitations, aggressive or inappropriate behaviour | 2.5 h |

Next to the care-need based assessment detailed above, about 5% of the care allowance cases are diagnosis-based. These include paraplegia, bilateral leg amputation, genetic muscle dystrophy or high-level visual impairment, blindness, and combined deaf- and blindness. ALTCA=Austrian long-term care allowance.

Supplementary Table 2: Requirement and amount of ALTCA by level

| Level | Requirement | Amount (in €) |
| --- | --- | --- |
| 1 | >2.2 h of care need per day | 192 |
| 2 | >3.2 h of care need per day | 354 |
| 3 | >4.0 h of care need per day | 552 |
| 4 | >5.3 h of care need per day | 827 |
| 5 | >6.0 h of care need per day and extraordinary care effort needed | 1,124 |
| 6 | >6.0 h of care need per day and care is required during day and night | 1,569 |
| 7 | >6.0 h of care need day and no coordinated movement of limbs possible | 2,062 |

Next to the care-need based assessment detailed above, about 5% of the care allowance cases are diagnosis-based. These include level 3 care allowance for paraplegia, bilateral leg amputation, genetic muscle dystrophy or high-level visual impairment, level 4 care allowance for blindness, and level 5 care allowance for combined deaf- and blindness. Amount in Euro was rounded to whole numbers. ALTCA=Austrian long-term care allowance.

Supplementary Figure 2: Distribution of the number of months of ATLCA received during the last 10 years (120 months) of life by sex


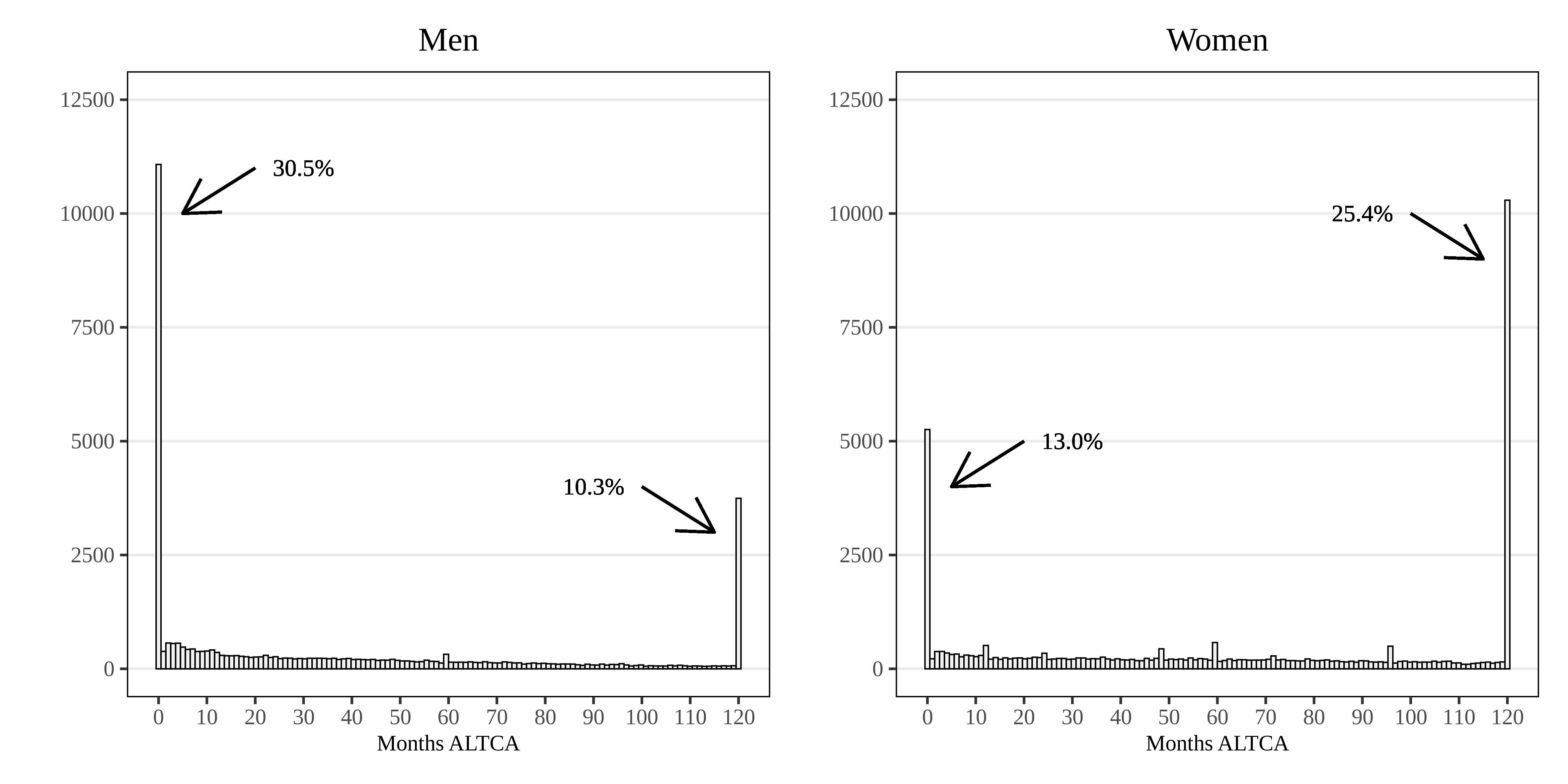


Total number of decedents aged 65 years and above=76,781, men=36,330, women=40,451. ALTCA=Austrian long-term care allowance.

Supplementary Table 3: Estimated months ALTCA was received for men and women by cause of death (taxonomic approach)

| Cause of death (ICD-10 codes) | Men | Women |
| --- | --- | --- |
| A00-B00 | 42.3 (37.4, 47.6) | 69.5 (64.6, 73.8) |
| C00-D49 | 27.6 (26.8, 28.4) | 53.5 (52.2, 54.7) |
| E00-E89 | 48.6 (46.3, 50.9) | 79.6 (77.7, 81.4) |
| F00-F99 | 50.1 (47.6, 52.7) | 74.8 (72.9, 76.6) |
| G00-G99 | 56.8 (54.6, 58.9) | 80.1 (78.3, 81.8) |
| I00-I99 | 37.4 (36.7, 38.2) | 70.4 (69.7, 71.2) |
| J00-J99 | 46.7 (45.0, 48.5) | 75.9 (74.1, 77.7) |
| K00-K95 | 37.2 (24.6, 39.9) | 68.6 (65.7, 71.3) |
| N00-N99 | 45.4 (42.5, 48.5) | 73.0 (70.7, 75.3) |
| R00-R99 | 27.8 (25.1, 30.6) | 59.8 (56.2, 63.5) |
| U00-U85 | 40.7 (39.1, 42.3) | 76.5 (74.9, 78.1) |
| V00-Y99 | 30.6 (28.7, 32.6) | 63.4 (60.6, 66.1) |
| Other | 43.9 (38.1, 50.2) | 74.7 (70.3, 78.8) |

Estimates based on marginal effects of zero-one-inflated beta regression models. Estimates refer to older adults of median age at death. ALTCA=Austrian long-term care allowance, A00-B00=infectious/parasitic diseases, C00-D48=neoplasms, E00-E90=endocrine/nutritional/metabolic, F00-F99=mental/behavioural, G00-G99=nervous system, H00-H95=eye/ear, I00-I99=circulatory system, J00-J99=respiratory system, K00-K93=digestive system, N00-N99=genitourinary system, R00-R99=non-classified, V01-Y98=external causes of death.

Supplementary Table 4: Estimated months ALTCA was received for men and women by cause of death (frequency-based approach)

| Cause of death (ICD-10 codes) | Men | Women |
| --- | --- | --- |
| C18.9 | 29.3 (25.9, 33.0) | - |
| C25.9 | 18.9 (16.8, 21.4) | 44.2 (39.7, 48.8) |
| C34.9 | 25.6 (23.7, 27.5) | 53.7 (49.9, 57.3) |
| C50.9 | - | 59.9 (56.5, 62.4) |
| C61 | 30.8 (28.9, 32.8) | - |
| F03 | 52.5 (49.2, 55.8) | 75.0 (73.0, 77.0) |
| G20 | 60.5 (57.2, 63.7) | 84.6 (81.3, 87.7) |
| G30.9 | - | 79.6 (76.6, 82.5) |
| I10 | - | 71.3 (67.0, 75.6) |
| I11.0 | - | 72.0 (68.6, 75.2) |
| I11.9 | - | 74.0 (70.5, 77.4) |
| I12.0 | - | 73.8 (69.1, 78.3) |
| I21.9 | 26.1 (24.2, 28.3) | 66.9 (63.7, 69.9) |
| I25.1 | 39.8 (38.3, 41.5) | 73.6 (71.9, 75.2) |
| I25.5 | 36.3 (32.9, 40.0) | 69.7 (65.4, 73.9) |
| I25.8 | 37.7 (34.4, 41.3) | 73.9 (70.0, 77.6) |
| I35.0 | - | 64.0 (59.4, 68.3) |
| I42.9 | - | 71.8 (67.2, 76.1) |
| I48.9 | 45.6 (41.7, 49.5) | 71.6 (68.5, 74.5) |
| I50.9 | 43.0 (39.6, 46.6) | 72.6 (69.8, 75.2) |
| I51.9 | - | 72.8 (68.8, 76.8) |
| I64 | 48.3 (44.4, 52.4) | 70.5 (67.5, 73.4) |
| J44.0 | 49.3 (46.8, 51.7) | - |
| J44.9 | - | 77.9 (74.6, 81.0) |
| N18.9 | - | 72.1 (67.7, 76.4) |
| R54 | - | 74.2 (69.8, 78.3) |
| R99 | 16.7 (13.9, 19.8) | - |
| U071 | 40.4 (38.8, 42.1) | 76.3 (74.6, 77.9) |
| X59.0 | - | 69.7 (65.9, 73.4) |
| Other | 36.9 (36.3, 37.5) | 65.9 (65.2, 66.7) |

Estimates based on marginal effects of zero-one-inflated beta regression models. Estimates refer to older adults of median age at death. ALTCA=Austrian long-term care allowance, C18.9=colon cancer, C25.9=pancreas cancer, C34.9=lung cancer, C50.9=breast cancer, C61=prostate cancer, F03=unspecified dementia, G20=Parkinson’s disease, G30.9=Alzheimer’s disease, I10=hypertension, hypertensive heart disease with heart failure, I11.9=hypertensive heart disease without heart failure, I12.0=hypertensive renal disease with renal failure, I21.9=acute myocardial infarction, I25.1=atherosclerotic heart disease, I25.5=ischaemic cardiomyopathy, I25.8=other forms of ischaemic heart disease, I35.0=aortic stenosis, unspecified cardiomyopathy, I48.9=atrial fibrillation, I50.9=unspecified heart failure, I64=stroke, I440=chronic obstructive pulmonary disease with acute lower respiratory infection, I44.9=unspecified chronic obstructive pulmonary disease, N18.9=chronic kidney disease, R54=senility, R99=other ill-defined or unspecific cause of death, U07.1=COVID-19, virus identified, X59.0=fracture with unknown or unspecified circumstance.

Supplementary Figure 3: Estimated months of receipt of ALTCA before death by sex, age, and cause of death, adjusted for marital status and education


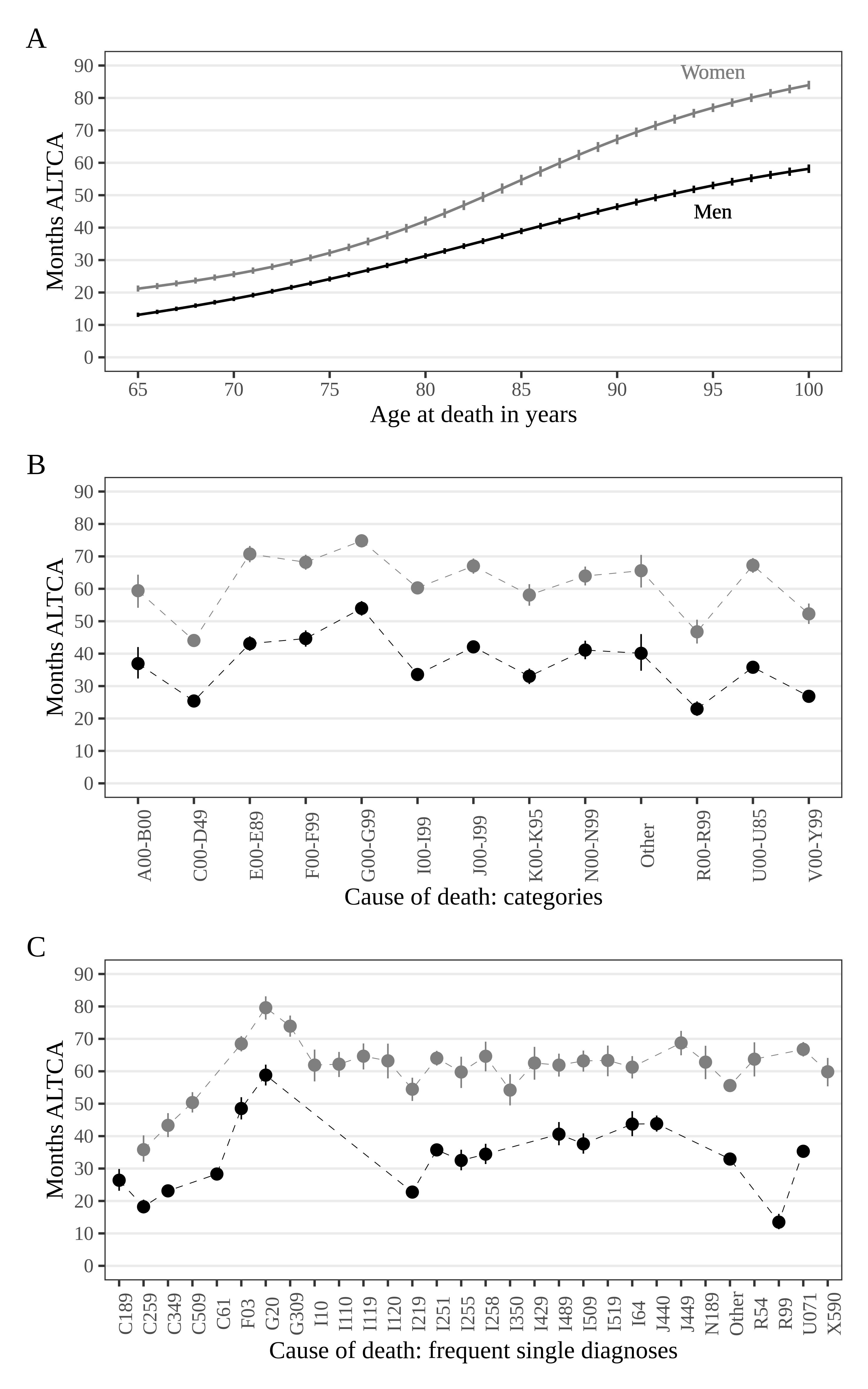


Estimates based on marginal effects of zero-one-inflated beta regression models based on 76,781 observations. Lines (panel A) and points (panels B-C) refer to point estimates, vertical lines (panel A-C) show 95% credible intervals. Estimates refer to older adults who died from cardiovascular diseases (ICD-10: I00-I99) (Panel A), at median age at death (Panels B-C) and who were married and had completed vocational education (Panels A-C). ALTCA=Austrian long-term care allowance, A00-B00=infectious/parasitic diseases, C00-D48=neoplasms, E00-E90=endocrine/nutritional/metabolic, F00-F99=mental/behavioral, G00-G99=nervous system, I00-I99=circulatory system, J00-J99=respiratory system, K00-K93=digestive system, N00-N99=genitourinary system, R00-R99=non-classified, U00-U95=provisional assignment of new diseases of uncertain etiology or emergency use, V00-Y99=external causes of death. C189=colon cancer, C259=pancreas cancer, C349=lung cancer, C50.9=breast cancer, C61=prostate cancer, F03=unspecified dementia, G20=Parkinson’s disease, G309=Alzheimer’s disease, I10=hypertension, hypertensive heart disease with heart failure, I119=hypertensive heart disease without heart failure, I120=hypertensive renal disease with renal failure, I21.9=acute myocardial infarction, I251=atherosclerotic heart disease, I255=ischemic cardiomyopathy, I258=other forms of ischemic heart disease, I350=aortic stenosis, I429=unspecified cardiomyopathy, I489=atrial fibrillation, I509=unspecified heart failure, I64=stroke, J440=chronic obstructive pulmonary disease with acute lower respiratory infection, J449=unspecified chronic obstructive pulmonary disease, N18.9=chronic kidney disease, R54=senility, R99=other ill-defined or unspecific cause of death, U071=COVID-19, virus identified, X590=fracture with unknown or unspecified circumstance.

Supplementary Table 5: Estimated probability to not receive ALTCA for men and women by cause of death (taxonomic approach)

| Cause of death (ICD-10 codes) | Men | Women |
| --- | --- | --- |
| A00-B00 | 0.29 (0.25, 0.35) | 0.09 (0.07, 0.12) |
| C00-D49 | 0.29 (0.27, 0.29) | 0.09 (0.08, 0.09) |
| E00-E89 | 0.20 (0.18, 0.22) | 0.05 (0.04, 0.06) |
| F00-F99 | 0.10 (0.09, 0.12) | 0.01 (0.01, 0.02) |
| G00-G99 | 0.06 (0.05, 0.07) | 0.01 (0.01, 0.01) |
| I00-I99 | 0.32 (0.31, 0.33) | 0.09 (0.08, 0.09) |
| J00-J99 | 0.18 (0.17, 0.20) | 0.04 (0.04, 0.05) |
| K00-K95 | 0.29 (0.27, 0.32) | 0.10 (0.08, 0.11) |
| N00-N99 | 0.14 (0.12, 0.17) | 0.04 (0.03, 0.05) |
| R00-R99 | 0.56 (0.53, 0.59) | 0.24 (0.21, 0.26) |
| U00-U85 | 0.33 (0.31, 0.34) | 0.06 (0.05, 0.07) |
| V00-Y99 | 0.43 (0.40, 0.45) | 0.14 (0.12, 0.16) |
| Other | 0.24 (0.53, 0.59) | 0.05 (0.04, 0.07) |

Estimates based on logistic regression models. Estimates refer to older adults of median age at death. ALTCA=Austrian long-term care allowance, A00-B00=infectious/parasitic diseases, C00-D48=neoplasms, E00-E90=endocrine/nutritional/metabolic, F00-F99=mental/behavioural, G00-G99=nervous system, H00-H95=eye/ear, I00-I99=circulatory system, J00-J99=respiratory system, K00-K93=digestive system, N00-N99=genitourinary system, R00-R99=non-classified, V01-Y98=external causes of death.

Supplementary Table 6: Estimated probability to not receive ALTCA for men and women by cause of death (prevalence-based approach)

| Cause of death (ICD-10 codes) | Men | Women |
| --- | --- | --- |
| C18.9 | 0.29 (0.24, 0.33) | - |
| C25.9 | 0.35 (0.31, 0.39) | 0.11 (0.09, 0.12) |
| C34.9 | 0.32 (0.30, 0.34) | 0.10 (0.08, 0.11) |
| C50.9 | - | 0.08 (0.07, 0.10) |
| C61 | 0.23 (0.20, 0.25) | - |
| F03 | 0.04 (0.02, 0.06) | 0.01 (0.01, 0.01) |
| G20 | 0.04 (0.02, 0.05) | 0.00 (0.00, 0.01) |
| G30.9 | - | 0.00 (0.00, 0.01) |
| I10 | - | 0.07 (0.05, 0.10) |
| I11.0 | - | 0.07 (0.05, 0.09) |
| I11.9 | - | 0.08 (0.06, 0.10) |
| I12.0 | - | 0.05 (0.03, 0.08) |
| I21.9 | 0.51 (0.49, 0.54) | 0.16 (0.14, 0.18) |
| I25.1 | 0.27 (0.25, 0.29) | 0.07 (0.06, 0.08) |
| I25.5 | 0.27 (0.23, 0.31) | 0.09 (0.06, 0.11) |
| I25.8 | 0.30 (0.26, 0.34) | 0.06 (0.04, 0.08) |
| I35.0 | - | 0.07 (0.05, 0.10) |
| I42.9 | - | 0.07 (0.05, 0.09) |
| I48.9 | 0.20 (0.17, 0.24) | 0.06 (0.05, 0.08) |
| I50.9 | 0.23 (0.20, 0.27) | 0.06 (0.04, 0.08) |
| I51.9 | - | 0.06 (0.04, 0.08) |
| I64 | 0.19 (0.16, 0.23) | 0.06 (0.05, 0.08) |
| J44.0 | 0.17 (0.15, 0.19) | - |
| J44.9 | - | 0.04 (0.03, 0.06) |
| N18.9 | - | 0.03 (0.02, 0.05) |
| R54 | - | 0.06 (0.04, 0.09) |
| R99 | 0.74 (0.70, 0.77) | - |
| U071 | 0.33 (0.31, 0.35) | 0.06 (0.06, 0.07) |
| X59.0 | - | 0.07 (0.05, 0.09) |
| Other | 0.28 (0.27, 0.28) | 0.09 (0.09, 0.10) |

Estimates based on logistic regression models. Estimates refer to older adults of median age at death. ALTCA=Austrian long-term care allowance, C18.9=colon cancer, C25.9=pancreas cancer, C34.9=lung cancer, C50.9=breast cancer, C61=prostate cancer, F03=unspecified dementia, G20=Parkinson’s disease, G30.9=Alzheimer’s disease, I10=hypertension, hypertensive heart disease with heart failure, I11.9=hypertensive heart disease without heart failure, I12.0=hypertensive renal disease with renal failure, I21.9=acute myocardial infarction, I25.1=atherosclerotic heart disease, I25.5=ischaemic cardiomyopathy, I25.8=other forms of ischaemic heart disease, I35.0=aortic stenosis, unspecified cardiomyopathy, I48.9=atrial fibrillation, I50.9=unspecified heart failure, I64=stroke, I440=chronic obstructive pulmonary disease with acute lower respiratory infection, I44.9=unspecified chronic obstructive pulmonary disease, N18.9=chronic kidney disease, R54=senility, R99=other ill-defined or unspecific cause of death, U07.1=COVID-19, virus identified, X59.0=fracture with unknown or unspecified circumstance.

Supplementary Figure 4: Probability to not receive ALTCA before death by sex, age, and cause of death, adjusted for marital status and education


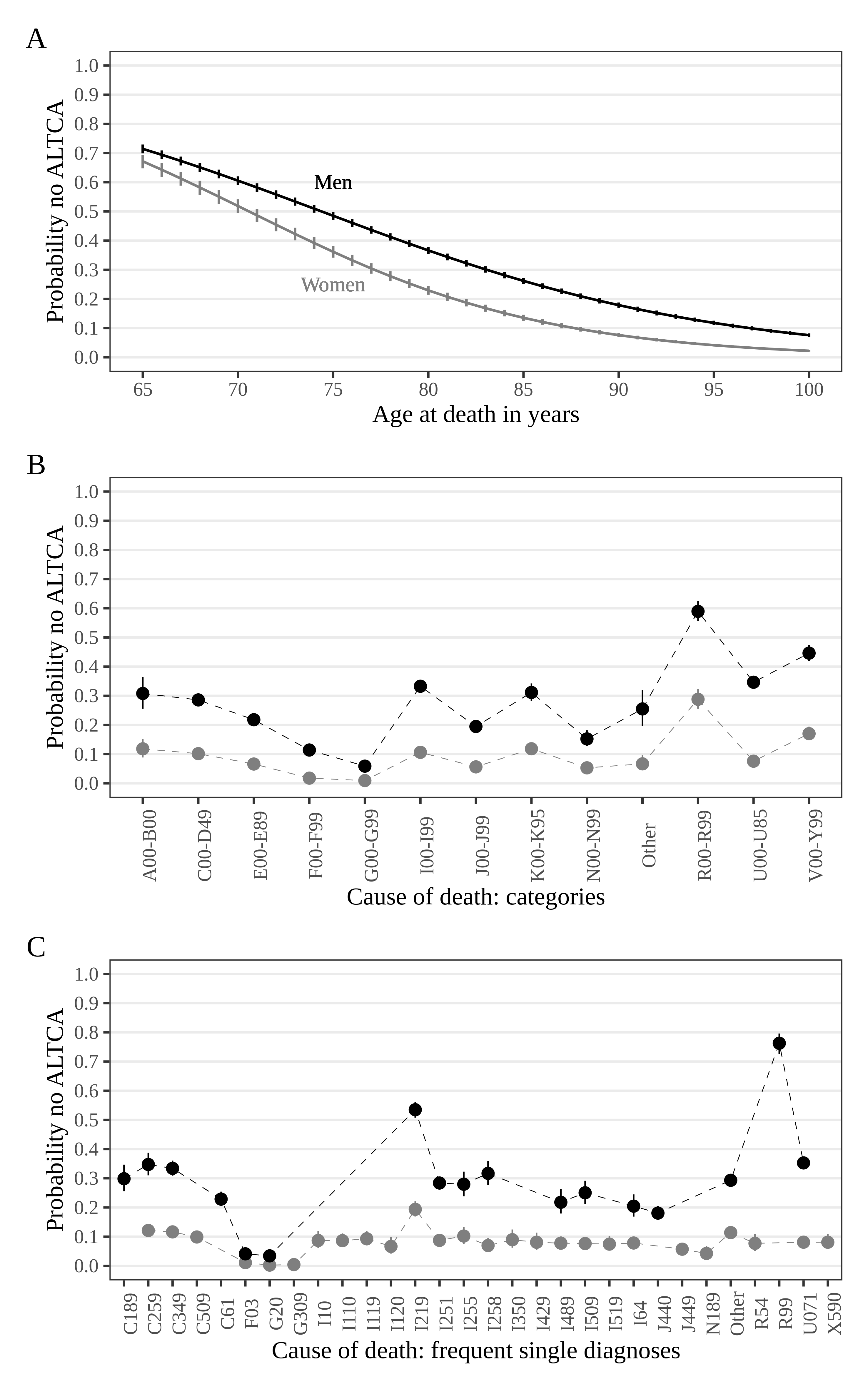


Estimates based on marginal effects of logistic regression models based on 76,781 observations. Lines (panel A) and points (panels B-C) refer to point estimates, vertical lines (panel A-C) show 95% credible intervals. Estimates refer to older adults who died from cardiovascular diseases (ICD-10: I00-I99) (Panel A), at median age at death (Panels B-C), and who were married and had completed vocational education (Panels A-C). ALTCA=Austrian long-term care allowance, A00-B00=infectious/parasitic diseases, C00-D48=neoplasms, E00-E90=endocrine/nutritional/metabolic, F00-F99=mental/behavioral, G00-G99=nervous system, I00-I99=circulatory system, J00-J99=respiratory system, K00-K93=digestive system, N00-N99=genitourinary system, R00-R99=non-classified, U00-U95=provisional assignment of new diseases of uncertain etiology or emergency use, V00-Y99=external causes of death. C189=colon cancer, C259=pancreas cancer, C349=lung cancer, C50.9=breast cancer, C61=prostate cancer, F03=unspecified dementia, G20=Parkinson’s disease, G309=Alzheimer’s disease, I10=hypertension, hypertensive heart disease with heart failure, I119=hypertensive heart disease without heart failure, I120=hypertensive renal disease with renal failure, I21.9=acute myocardial infarction, I251=atherosclerotic heart disease, I255=ischemic cardiomyopathy, I258=other forms of ischemic heart disease, I350=aortic stenosis, I429=unspecified cardiomyopathy, I489=atrial fibrillation, I509=unspecified heart failure, I64=stroke, J440=chronic obstructive pulmonary disease with acute lower respiratory infection, J449=unspecified chronic obstructive pulmonary disease, N18.9=chronic kidney disease, R54=senility, R99=other ill-defined or unspecific cause of death, U071=COVID-19, virus identified, X590=fracture with unknown or unspecified circumstance.
